# The Role of METTL3-Mediated N6-Methyladenosine (m6A) of JPH2 mRNA in Cyclophosphamide-Induced Cardiotoxicity

**DOI:** 10.3389/fcvm.2021.763469

**Published:** 2021-11-08

**Authors:** Min Zhu, Yangong Liu, Yuanxiu Song, Shiqin Zhang, Chengwen Hang, Fujian Wu, Xianjuan Lin, Zenghui Huang, Feng Lan, Ming Xu

**Affiliations:** ^1^Department of Cardiology and Institute of Vascular Medicine, NHC Key Laboratory of Cardiovascular Molecular Biology and Regulatory Peptides, Key Laboratory of Molecular Cardiovascular Science, Ministry of Education, Beijing Key Laboratory of Cardiovascular Receptors Research, Peking University Third Hospital, Beijing, China; ^2^State Key Laboratory of Cardiovascular Disease, National Center for Cardiovascular Diseases, Fuwai Hospital, Key Laboratory of Application of Pluripotent Stem Cells in Heart Regeneration, Chinese Academy of Medical Sciences and Peking Union Medical College, Beijing, China; ^3^Beijing Lab for Cardiovascular Precision Medicine, Anzhen Hospital, Capital Medical University, Beijing, China; ^4^Key Laboratory of Genetic Network Biology, Institute of Genetics and Developmental Biology, Chinese Academy of Sciences (CAS), Beijing, China; ^5^State Key Laboratory of Natural and Biomimetic Drugs, Peking University, Beijing, China

**Keywords:** cyclophosphamide, cardiotoxicity, JPH2, m6A methylation, METTL3, cardiomyocyte

## Abstract

Cyclophosphamide (CYP)-induced cardiotoxicity is a common side effect of cancer treatment. Although it has received significant attention, the related mechanisms of CYP-induced cardiotoxicity remain largely unknown. In this study, we used cell and animal models to investigate the effect of CYP on cardiomyocytes. Our data demonstrated that CYP-induced a prolonged cardiac QT interval and electromechanical coupling time courses accompanied by JPH2 downregulation. Moreover, N6-methyladenosine (m6A) methylation sequencing and RNA sequencing suggested that CYP induced cardiotoxicity by dysregulating calcium signaling. Importantly, our results demonstrated that CYP induced an increase in the m6A level of JPH2 mRNA by upregulating methyltransferases METTL3, leading to the reduction of JPH2 expression levels, as well as increased field potential duration and action potential duration in cardiomyocytes. Our results revealed a novel mechanism for m6A methylation-dependent regulation of JPH2, which provides new strategies for the treatment and prevention of CYP-induced cardiotoxicity.

## Introduction

Although improved treatments have been effective in increasing the survival of patients with tumors, an increase in the number of side effects of cancer treatment have led to mortality (1, 2). Tumor therapy-induced cardiotoxicity as a common side effect has received increasing attention. Many countries and regions have issued relevant practice guidelines for cardiovascular toxicity induced by cancer treatments (3). Both conventional chemotherapies and targeted drug therapies reportedly induce cardiovascular toxicity events. One traditional antineoplastic agent, cyclophosphamide (CYP), is employed in the treatment of various cancers, including breast, lymphoid, and hematologic malignancies (4). Up to 28% of patients who received a high dose of CYP suffered from cardiac arrhythmias (3) and even heart failure (5). Further, CYP is widely used in the treatment of other diseases, such as refractory neuromyelitis optica spectrum disorder (200 mg/kg) (6) and rapidly progressive systemic sclerosis (300 mg/kg) (7), all of reportedly cause severe cardiotoxicity. Even in the clinic, oral administration of a low dose (50 or 100 mg/day) for systemic sclerosis or lupus erythematosus for 1 week has caused cardiac electrical alteration (prolonged QT interval) in some patients. However, little is known about the mechanism underlying CYP-related cardiovascular toxicity. In particular, CYP has often been used in combination with other antineoplastic agents, including anthracyclines, docetaxel, and trastuzumab. This has led to difficulty in assessing the contribution of CYP in multidrug schemes (8).

CYP and other alkylating agents are the most common types of DNA damaging agents used in the treatment of various cancers. Alkylating agents exhibit pharmacological toxicity by adding methyl and other hydrocarbon groups to the DNA bases, resulting in base mutations, pair mismatches, and eventually fatal DNA cytotoxicity (9). The pharmacological mechanism is fatal to rapidly proliferating tumor cells. However, the cardiac cytotoxicity induced by alkylating agents is rarely discussed for non-proliferating cardiomyocytes. Because alkylating agents adduct DNA bases (A, T, G, and C) to induce DNA methylation (9), alkylating agents might affect RNA methylation. N6-adenosine methylation (m6A) of RNA transcripts is the most prevalent RNA modification (10). This modification regulates RNA stability (11), gene expression (12), mRNA alternative splicing (13), embryonic and stem cell differentiation (13–15), and various diseases including cancer (16) and cardiac dysfunctions (11, 17). Hence, we hypothesized that CYP induces cardiotoxicity through RNA m6A modification.

We treated rat neonatal cardiomyocytes (NRCMs), human embryonic stem cell-derived cardiomyocytes (hESCs-CMs), and a rat model with CYP to explore solutions for this problem. This was followed by combining action and field potential detections, RNA sequencing, and RNA m6A methylation analysis to explore the toxicity mechanism underlying CYP-induced cardiac electrical and mechanical alterations. Our results may provide drug targets and preventive measures for treating CYP-induced cardiotoxicity.

## Materials and Methods

### Animals

All Sprague-Dawley (SD) rats in this study were purchased from Beijing Vital River Laboratory Animal Technology Company (Beijing, China). Twelve 8-week-old male SD rats with a mean weight of 273.7 ± 3.2 g were randomized into two groups: six rats were subjected to saline (Double Crane Pharmaceutical Co. Ltd, Wuhan, China) peritoneal injection (vehicle group), whereas six rats were intraperitoneally injected with CYP (Jiangsu Hengrui Medicine Co., Ltd. Lianyungang, China) at a dose of 100 mg/kg (CYP treatment group). Echocardiography (echo) and electrocardiography (ECG) were performed at different time points (0, 1, and 3 days).

### *In vivo* ECG Recording

Continuous recordings of heart rate were obtained with a surface ECG. Rats were anesthetized with 3% isoflurane and were subsequently fixed on a wooden board. ECG recording was performed using the limb lead. Three electrodes on an ECG monitor were inserted into the subcutaneous tissues of the rats' left and right shoulders and the right hind leg. The signal was amplified and recorded on a personal computer using an ECG Processor (EP-2B, Softron Beijing Incorporated, China) and stored on a data acquisition program (SP2006, Softron Beijing Incorporated, China).

### ECG and Electromechanical Coupling Time Measurement

ECG measurement was performed as described previously (18). ECG was performed using a Vevo 2,100 system (FUJIFILM VisualSonics, Canada), and the cardiac dimensions and functional parameters were measured. The tissue Doppler imaging (TDI) echo combined with ECG was used to measure the electromechanical coupling time at the lateral wall of the left ventricle as described previously (19).

### Neonatal Rat Cardiac Myocytes Culture

NRCMs were isolated from newborn SD rats aged 1–2 days as described previously (20). These isolated NRCMs were grown in Dulbecco's modified Eagle's medium supplemented with 10% fetal bovine serum and 100 U/ml penicillin/streptomycin, and maintained at 37°C in 5% CO_2_.

### Cardiac Differentiation of Human Embryonic Stem Cells (hESC)

H9 human embryonic stem cells were purchased from the Beijing Cellapy Biological Technology Company (Cellapy, China). H9 cells were cultured and differentiated into cardiomyocytes following previously described procedures (21). In brief, H9 cells were cultured on 35-mm dishes (Corning, USA) with PSCeasy hESC culture medium (Cellapy, Beijing, China). Cells were cultured to reach ~90% confluency and differentiated into ESC-CMs using a chemical method as described previously (22). Immunofluorescent staining with primary antibodies against TNNT2 (Santa Cruz, USA) and α-actinin (Abcam, UK) validated the purity of human cardiomyocytes.

### Immunofluorescence

Cells were cultured on glass slides, washed with PBS three times, fixed in 4% paraformaldehyde for 5 min, and then permeabilized with PBS containing 0.5% Triton X-100 (Sigma, USA) for 10 min. After 1 h of blocking with 5% BSA (Amresco, USA), the slides were incubated with primary antibodies followed by incubation with secondary antibodies. After the slides were washed, they were studied with a confocal fluorescence imaging microscope (DMI 4000B, Leica, Germany). The primary and secondary antibodies and their appropriate dilutions are listed in Supplementary Table 1.

### Microelectrode Array (MEA) Analysis

MEA recording in cardiomyocytes was performed as described previously (23). In brief, 2 × 10^4^ cells were plated on CytoView MEA plates (Axion Biosystems, USA) pre-coated with 5% matrigel, followed by treatment with CYP at different concentrations (0 and 500 μmoL/L). The experimental data were acquired using a Maestro EDGE (Axion Biosystems, USA) according to the MEA operation manual.

### RNA Extraction and Quantitative Real-Time PCR

Total RNA was extracted from NRCMs using the TRIzol reagent (Invitrogen) and subjected to reverse transcription (RT) and real-time PCR. The primers used are listed in Supplementary Table 2. RT was performed using a 2,720 Thermal Cycler (Applied Biosystems, USA). Real-time PCR was performed using a QuantStudio 3 apparatus (Applied Biosystems, USA).

### Western Blot Analysis

Proteins were extracted from cells in RIPA lysis buffer (Solarbio, China) containing 1 mmol/L PMSF (Solarbio, China) and protease inhibitor cocktail (Bimake, China) for western blot analysis. In total, 50 μg of protein were subjected to SDS-PAGE, transferred to a PVDF membrane (Millipore, USA), and incubated with the primary antibodies. These primary antibodies and their appropriate dilutions are listed in Supplementary Table 1. The membrane was then incubated with HRP-conjugated goat anti-mouse IgG (1: 2000, ZSGB-BIO) or HRP-conjugated goat anti-rabbit IgG (1: 2000, ZSGB-BIO). GAPDH was used as a control. Protein levels were determined using the Immobilon® Western Chemiluminescent HRP substrate (Millipore, UK).

### Cell Treatments

In the CYP treatment assays, 250, 500, and 750 μmol/L of CYP (Selleck, USA) were added to the cell complete culture medium for 2 or 4 days for NRCMs and for 2 or 5 days for hESCs-CMs. To impede the expression of METTL3 in cardiomyocytes, adenoviruses harboring the specific small interference RNA (siRNA) sequences of METTL3 were used individually to infect cardiomyocytes at an optimized MOI for 24 h, followed by treatment with 500 μmol/L CYP for an additional 24 h as the MEA assay. The siRNA and negative control (NC) sequences used are listed in Supplementary Table 2.

### RNA m6A Dot Blot Assay

An RNA m6A dot blot assay was performed as previously described (24). In brief, 1.5 μg of total RNA was spotted onto a positively charged nylon-based membrane (GE Healthcare), blocked with 5% milk at room temperature for 2 h, and incubated with anti-m6A antibodies (1: 2000, Abcam) at 4 °C overnight and secondary antibodies (1: 3000, Abcam) at room temperature for 2 h. The same RNAs were spotted on the positively charged nylon-based membrane and stained with 0.02% methylene blue in 0.3M sodium acetate (pH 5.2), which ensured loading consistency among different samples.

### Methylated RNA Immune Precipitation (MeRIP) Sequencing

High throughput m6A sequencing was performed with the support of Kangchen Biotech (Shanghai, China). Briefly, total RNA was extracted from NRCMs treated with 500 μmol/L CYP or DMSO (solvent control) for 48 h, followed by random fragmentation to 100–150 nucleotides using RNA fragmentation reagents. Fragmented RNA was subjected to m6A antibody immunoprecipitation following the Magna MeRIP m6A kit protocol (17-10499, Merk Millipore, USA) as described previously (25). An RNA library from immunoprecipitated RNA and input RNA was created on an Illumina HiSeq platform. Differential m6A peaks (fold change ≥1.5 and *P* ≤ 0.05) between CYP and solvent controls were used for gene ontology (GO) enrichment and Kyoto Encyclopedia of Genes and Genomes (KEGG) analysis.

### Ca^2+^ Imaging

Ca^2+^ imaging in cardiomyocytes was performed as described previously (21). In brief, hESCs-CMs inoculate with the green fluorescent calcium-modulated protein (GCaMP) calcium sensor (H9-GCaMP-CMs) were seeded onto confocal dishes. Confocal microscope (Leica, TCS5 SP5, Germany) was used for intracellular calcium imaging. Spontaneous Ca^2+^ transients were recorded at 37°C and 5% CO_2_ according to the standard line-scan methods (26, 27). A total of 8,192 line scans were acquired for a duration of 8.192 s. The imaging results were analyzed using the Image J and Igor pro software.

### Statistical Analysis

All statistical analyses were conducted using the SPSS 20.0 software (IBM Corp., USA) and Graphpad Prism software (version 8.0, GraphPad Software Inc., USA). The data are expressed as the mean ± standard error (SE). A Student's *t*-test detected the differences between groups. *P* values of ≤ 0.05 were considered as statistically significant.

## Results

### CYP Increased the Field Potential Duration and Decreased the Contractile Amplitudes of Cardiomyocytes

**Figure 1:**
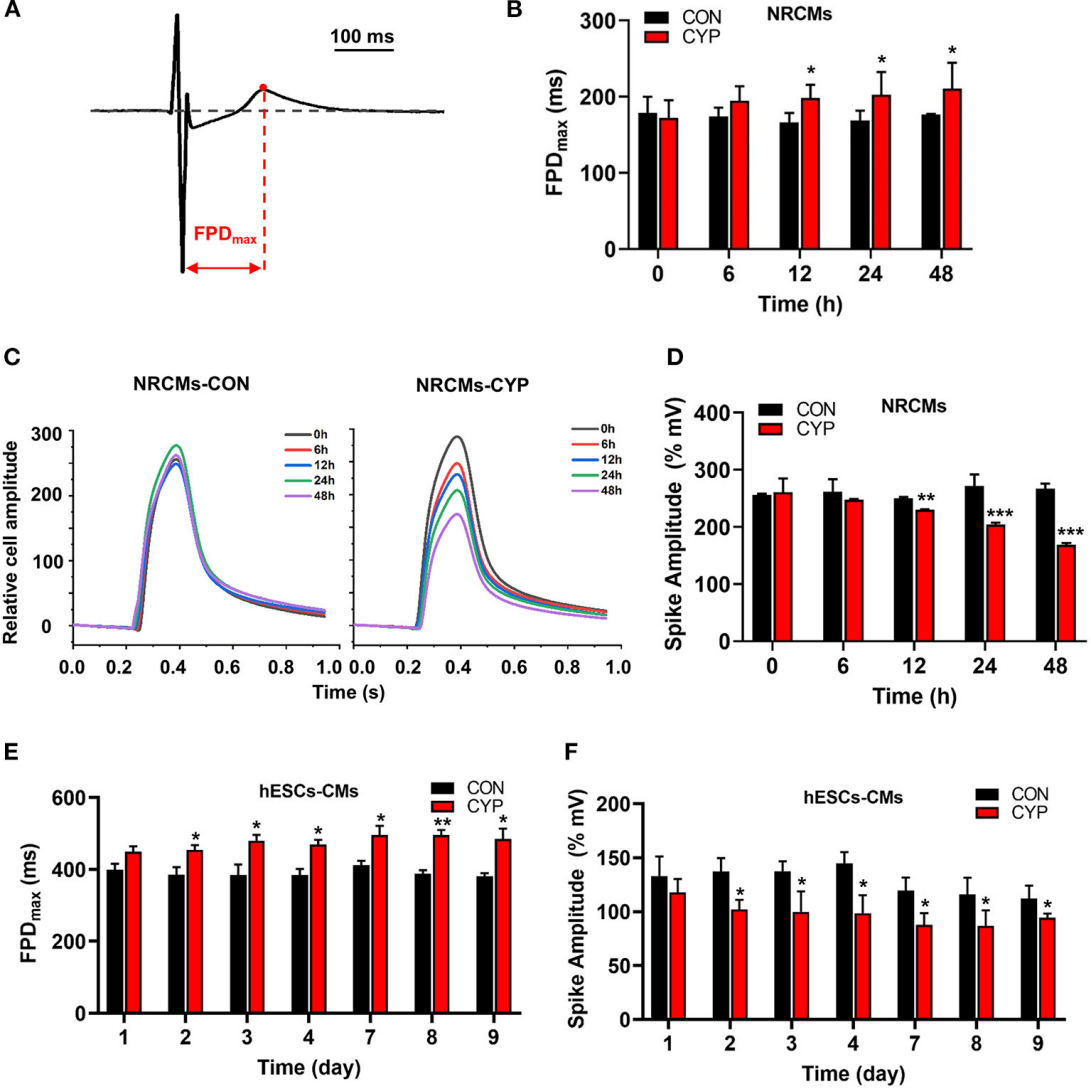
CYP increased the field potential duration (FPD) and decreased contractile amplitudes of cardiomyocytes. **(A)** Schematic of FPD of cardiomyocytes. **(B)** The FPD analysis of NRCMs with 500 μmol/L of CYP at 0, 6, 12, 24, and 48 h. **(C)** Representative images of the relative cell amplitude of NRCMs treated with solvent control (CON) or CYP. The data were shown as the mean of triplicate experimental wells. **(D)** The cell amplitude analysis of NRCMs treated with 500 μmol/L CYP at 0, 6, 12, 24, and 48 h. The FPD **(E)** and cell amplitude **(F)** analysis of hESCs-CMs treated with 500 μmol/L CYP at 1, 2, 3, 4,7,8, and 9 days. The data are shown as the mean±SE, *n* = 3. *P < 0.05, **P < 0.01, ***P < 0.001 vs. CON.

To clarify the cellular significance of CYP in cardiomyocytes, we first performed the CCK-8 assay to examine the effects of CYP on the viability of cardiomyocytes. The result confirmed that CYP had no significant effect on NRCMs viability (Supplementary Figure 1A). However, we observed that the levels of atrial natriuretic factor (ANP) and brain natriuretic peptide (BNP) had increased after NRCMs were treated with 500 μmoL/L CYP for 48 h (Supplementary Figures 1B,C). These results suggested that CYP induced slight cardiotoxicity, but did not affect cell viability. CYP was closely associated with cardiac arrhythmias related to QT prolongation and the acute and chronic toxicity of chemotherapy (3). A prolonged QT interval is an important monitoring indicator for myocyte toxicity caused by anticancer agents according to the guidelines issued by the International Conference on Harmonization of Technical Requirements for Registration of Pharmaceuticals for Human Use (28). Therefore, we seeded cardiomyocytes on multielectrode array (MEA) probes to evaluate the effect of CYP on myocardial electrophysiological properties. The time between depolarization and repolarization is the FPD (Figure 1A), which corresponds to the QT interval in an ECG. Compared with solvent control (CON), the FPD of NRCMs treated with CYP increased at 12, 24, and 48 h (Figure 1B). Meanwhile, the impedance of the CON cells showed no significant changes (Figure 1C). We observed that the impedance of NRCMs treated with CYP decreased with time (Figure 1D). These observations indicated that CYP negatively regulated the rhythm and contractility of cardiomyocytes.

Furthermore, we used human embryonic stem cell-derived cardiomyocytes (hESCs-CMs) (Supplementary Figure 1D) to evaluate the effect of CYP on cellular viability. Consistent with this finding in NRCMs, CYP had no significant effect on the viability of hESCs-CMs (Supplementary Figure 1E) but increased the RNA levels of ANP and BNP (Supplementary Figures 1F,G). Intriguingly, exposing hESCs-CMs to CYP (500 μmol/L) for 9 days resulted in a significant increase in FPD (Figure 1E) and reduction of contractile amplitudes (Figure 1F). These results supported QT interval prolongation and cardiac contractile dysfunction in rats.

### CYP-Induced Cardiac Electrical and Mechanical Alterations and Decreased Cardiac Contractile Function in Rats

**Figure 2:**
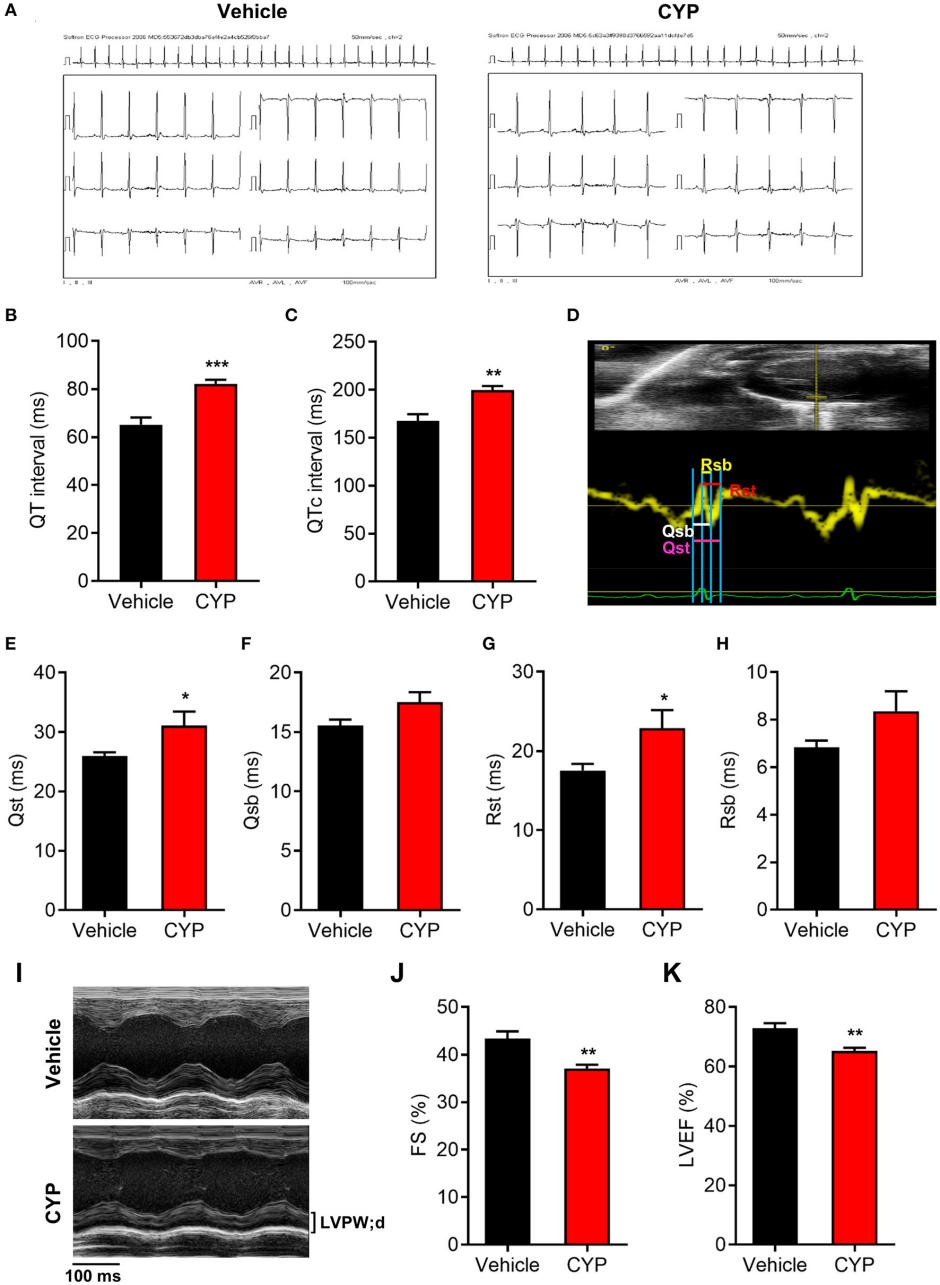
The effect of CYP on QT intervals, cardiac electromechanical coupling and cardiac function. Electrocardiogram recording **(A)** showing QT intervals **(B)** and QTc **(C)** prolongation in rats treated with CYP for 1 day. **(D)** Schematic of four time courses of cardiac electromechanical coupling in the lateral wall of the left ventricle of rats. Qsb time course is the duration from the onset of Q wave on ECG to the beginning of S wave. Qst time course is the duration from the onset of Q wave on ECG to the top of S wave. Rsb time course is the duration from the top of R wave on ECG to the beginning of S wave. Rst time course is the duration from the top of R wave on ECG to top of S wave. The TDI echo combined with ECG measurement revealed an increase in Qsb **(E)**, Qst **(F)**, Rsb **(G)**, and Rst **(H)** in CYP-treated rats compared with that in vehicle-treated rats. **(I)** Representative M-mode echocardiography in rats treated with vehicle and CYP for 1 day. Echocardiography revealed that fractional shortening (FS) **(J)** and left ventricular ejection fraction (LVEF) **(K)** decreased in CYP-treated rats as compared with that in vehicle-treated rats. The data are represented as mean ± SE, *n* = 6. *p < 0.05, **P < 0.01, ***P < 0.001 vs. vehicle.

To further investigate the effect of CYP on cardiac functions, we used intraperitoneally injected CYP to treat the rats with CYP at a dose of 100 mg/kg, which was converted from the clinical dose for treatment of cancer. *In vivo* ECG recording data (Figure 2A) showed QT interval prolongation in rats after CYP-treatment for 1 day compared with that in rats administered saline (82.17 ± 1.70 vs. 65.17 ± 3.02 ms, *P* < 0.001; Figure 2B). Corrected QT interval (QTc) prolongation also showed the same variation as QT prolongation (199.83 ± 4.03 vs. 167.67 ± 6.83 ms, *P* < 0.01; Figure 2C). Further, the prolonged QT and QTc would restore to preadministration levels after CYP-treatment for 3 days (Supplementary Figures 2A,B). These results were consistent with the clinical side effects of CYP.

Electromechanical coupling disturbances were closely related to the long QT syndrome (29, 30). Hence, we further explored the effect of CYP on cardiac electrical and mechanical alterations. Four electromechanical coupling time courses (Qsb, Qst, Rsb, and Rst) were measured with TDI echo combined with ECG (Figure 2D). The measurement results showed that four electromechanical coupling time courses in CYP-treated rats were longer than those in saline controls (Figures 2E–H), particularly in terms of Qst and Rst courses (*P* < 0.05, Figures 2E,G). Moreover, ultrasound echocardiography (Figure 2I) showed that the fractional shortening percentage (FS%; Figure 2J) and left ventricular ejection fraction (LVEF; Figure 2K) were significantly lower in rats after CYP-treatment for 1 day. These results suggested that CYP induces cardiac electrical and mechanical alterations and decreases the excitation-contraction (E-C) coupling efficiency, leading to cardiac contractile dysfunction. Consistent with the results of QT and QTc, the prolonged electromechanical coupling time courses would restore after CYP treatment for 3 days (Supplementary Figures 2C–F) and the decreased FS and LVEF induced by CYP also showed a regression in CYP-treated rats after 3 days (Supplementary Figures 2G,H).

### CYP-Induced the Decrease of JPH2 Expression in Cardiomyocytes

**Figure 3:**
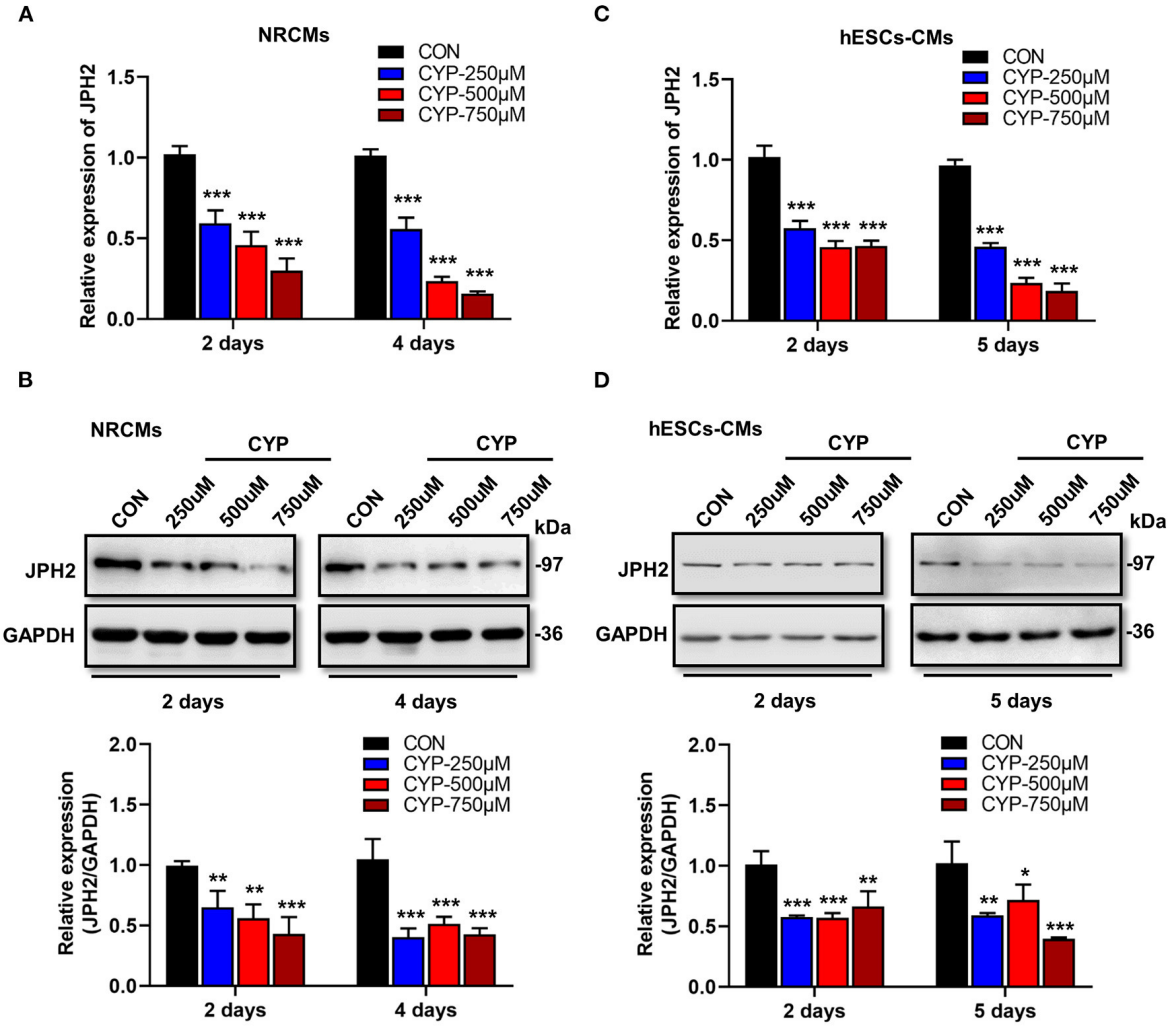
CYP induced the downregulation of JPH2 expression in cardiomyocytes. Real-time PCR **(A)** and western blot **(B)** analysis of JPH2 expression in NRCMs treated with CYP for 2 or 4 days. Real-time PCR **(C)** and western blot **(D)** analysis of JPH2 expression in hESCs-CMs treated with CYP for 2 or 5 days. The data are shown as the mean ± SE of three experiments. *P < 0.05, **P < 0.01, ***P < 0.001 vs. CON.

Previous studies demonstrated that junctophilin-2 (JPH2) that anchor the sarcoplasmic reticulum to T-tubules is the key regulator of Ca^2+^ influx between L-type Ca^2+^ channels (LCCs) and ryanodine receptors (RyRs) and E-C coupling in cardiomyocytes (31, 32), is reportedly associated with atrial fibrillation (33) and arrhythmias (34). Based on the phenomena observed in the above cell and animal experiments, we further investigated the effect of CYP on JPH2 expression in NRCMs and hESCs-CMs at different treatment time points. Notably, a dose-dependent reduction in JPH2 RNA and protein levels occurred in NRCMs treated with CYP for 2 or 4 days (Figures 3A,B). Similarly, different concentrations of CYP treatments decreased JPH2 both in RNA and protein levels at day 2 or 5 in hESCs-CMs (Figures 3C,D). Similarly, JPH2 downregulation occurred in heart tissues of rats treated with CYP (Supplementary Figure 3). These results suggested that CYP induced cardiac electrical and mechanical alterations and cardiac contractile dysfunction by decreasing the expression of JPH2.

To explore the underlying mechanisms involved in the suppression effects of CYP on JPH2 expression in cardiomyocytes, we further investigated the effect of CYP on miR-24 and miR-331 expressions, which were shown to inhibit the expression of JPH2 in our previous studies (31, 35). The real-time PCR analysis revealed that the expression of miR-24 and miR-331 did not significantly change in NRCMs after CYP treatment for 2 days (Supplementary Figures 4A,B). Therefore, it suggested that CYP decreased JPH2 expression through other transcriptional regulatory mechanisms.

### CYP-Induced Substantial m6A Changes in Cardiomyocytes

**Figure 4:**
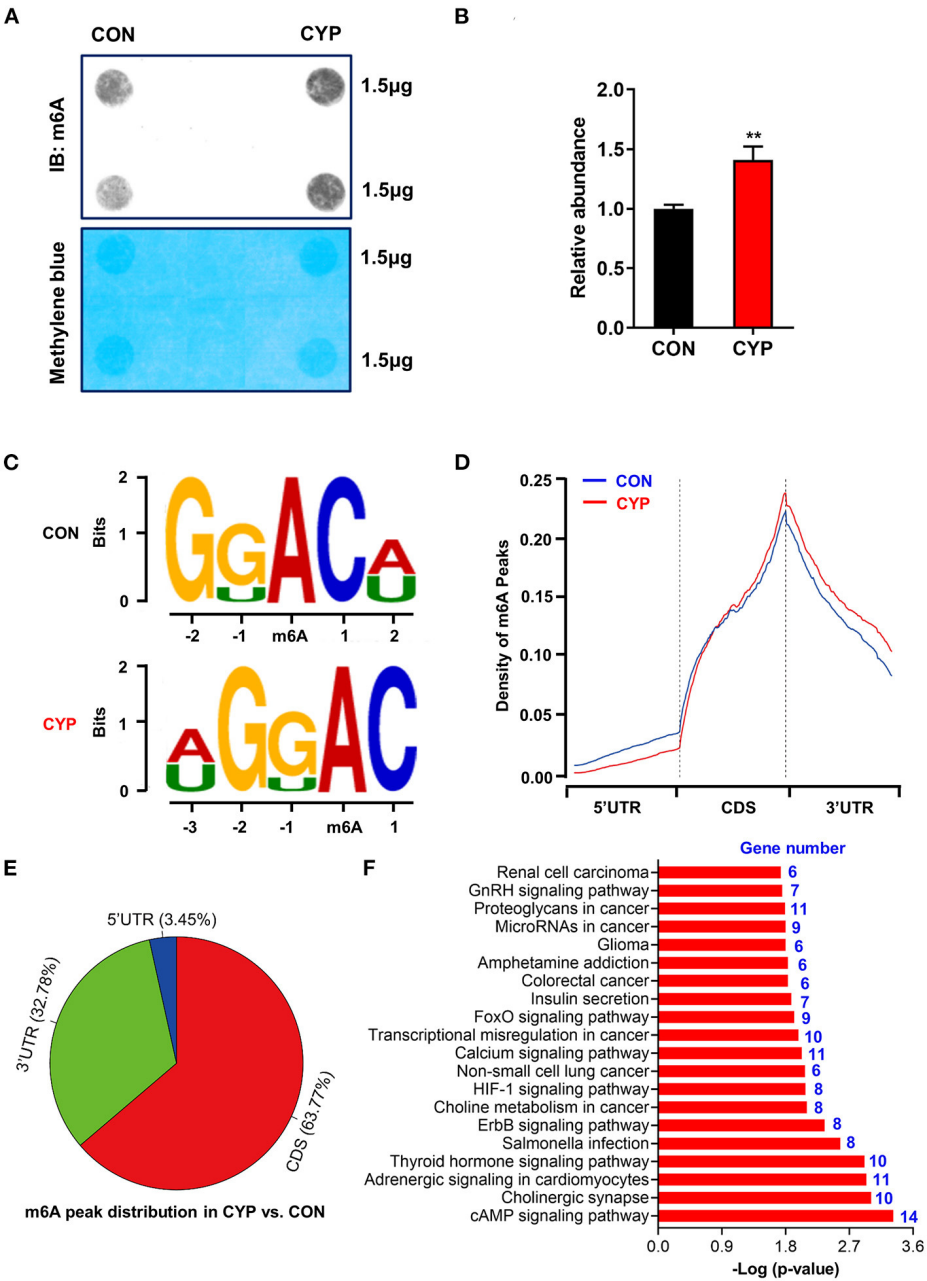
Overview of altered m6A-tagged transcripts landscape in NRCMs with or without CYP. **(A)** The m6A dot blot assay was conducted in NRCMs after treatment with CYP or solvent control (CON) for 2 days. Methylene blue staining was used as the loading control. **(B)** Quantitative analysis of m6A abundance in NRCMs treated with CYP or CON for 2 days. **(C)** Top sequence motif identified from MeRIP-seq peaks in control and CYP-treated NRCMs. **(D)** Metagene plots showing the region of average m6A peaks identified across all transcripts in NRCMs with solvent control or CYP. **(E)** Pie charts showing m6A peak distribution in DEGs between CYP and control (CON) groups. **(F)** The top twenty significantly enriched pathways of upregulation of m6A peaks transcripts. The data are shown as the mean ± SE from three separate experiments. *P < 0.05; **P < 0.01; ***P < 0.001 vs. CON.

N6-methyladenosine (m6A) is the most prevalent modification that widely exists in mRNAs, which is associated with post-transcriptional gene expression regulation (12), and mRNA stabiltity (36). We next investigated whether CYP plays an important role in m6A RNA methylation in NRCMs, considering that CYP can induce nucleic acid methylation. The m6A dot blot testing showed that total m6A levels significantly increased in NRCMs treated with CYP for 2 days (Figures 4A,B). Next, methylated RNA immune precipitation sequencing (MeRIP-seq) was performed to compare the global profiling of m6A target genes between solvent controls and CYP-treated NRCMs. As shown in Figure 4C, the sequence motif “GGAC” was highly enriched in m6A immunoprecipitated RNAs, consistent with the findings of previous studies (37, 38). We found 585 significantly increased m6A peaks distributed in 259 genes, whereas 277 genes had 548 statistically decreased m6A peaks in CYP-treated NRCMs relative to controls. Notably, we observed that reduced m6A peaks were mainly localized in the 5′ untranslated region (5′ UTR), whereas increased m6A peaks were distributed in the coding sequence (CDS) and 3′ untranslated region (3′ UTR; Figure 4D). The pie charts showed that these statistically differentially distributed m6A peaks were mainly noted in the CDS and 3′ UTR of genes in CYP-treated NRCMs regarding CON cells (Figure 4E). To explore the physiological and pathological significance of m6A modification after CYP treatment, we analyzed the KEGG pathway on the significantly altered m6A peaks. Our results showed that upregulated m6A peaks in the CYP-treated NRCMs were significantly related to the cAMP signaling pathway, adrenergic signaling in cardiomyocytes, calcium signaling pathway, GnRH signaling pathway and other dysregulation pathways in cancer (Figure 4F).

Furthermore, RNA sequencing was also performed on NRCMs treated with solvent control (CON) or CYP. Compared with CON, 369 genes were significantly downregulated, and 74 genes were upregulated in the CYP-treated group (Supplementary Figure 5A). The GO enrichment and KEGG analysis of the total DEGs showed that these DEGs were enriched in the NF-κB, TNF, and calcium signaling pathways (Supplementary Figures 5B,C). Remarkably, with the combined MeRIP-seq and RNA-seq results, we found upregulated m6A methylation sites in the 5′UTR and CDS of JPH2 mRNA, accompanied with the downregulation of JHP2 expression on the RNA level. These results suggested that CYP induces calcium signaling changes through JPH2 downregulation caused by increasing m6A modification.

### CYP-Induced Calcium Handling Abnormalities in hESCs-CMs

**Figure 5:**
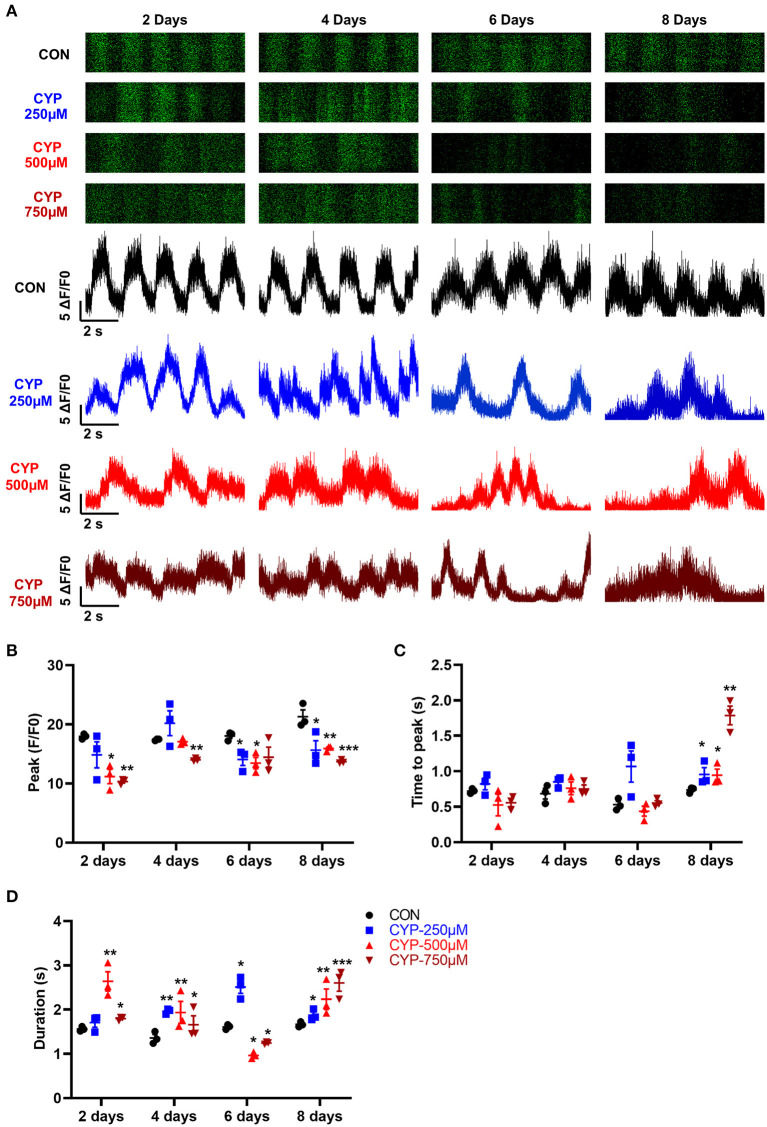
hESCs-CMs treated with CYP exhibited abnormal Ca^2+^ handling properties. **(A)** Representative line-scan images in H9-GCaMP cell-derived cardiomyocytes treated with different concentrations of CYP at 2, 4, 6 and 8 days. Quantification of peak **(B)**, time to peak **(C)**, and calcium transient duration **(D)** in CON and CYP-treated H9-GCaMP-CMs. The data are shown as the mean ± SE, *n* = 3. *P < 0.05, **P < 0.01, ***P < 0.001 vs. CON.

Calcium is a fundamental regulator of E-C coupling and electrophysiological signaling in cardiac myocytes (39). The above MeRIP-seq and RNA-seq results showed that the calcium signaling pathway played an important role in CYP-induced cardiotoxicity. We next verified and analyzed the Ca^2+^ handling properties of hESCs-CMs with CYP treatment by using H9-GCaMP derived cardiomyocytes (H9-GCaMP-CMs) (21). Compared with CON, hESCs-CMs treated with different concentrations of CYP (250, 500, and 750 μmoL/L) demonstrated significant Ca^2+^ transient irregularities, which were virtually absent in CON cells. As shown in Figure 5A, H9-GCaMP-CMs treated with 250 μmoL/L CYP showed no significant changes in the rhythm of Ca^2+^ transient release and reabsorption regarding CON on day 2. As the treatment time prolonged, the cardiomyocytes exhibited longer Ca^2+^ transient durations on day 4 and slower beating rate, lower Ca^2+^ release amplitude, and longer transient durations on days 6 and 8 (Figures 5B–D). We noted a similar pattern of changes in H9-GCaMP-CMs treated with 500 or 750 μmoL/L CYP at different time points. On days 2 and 4, compared with CON, CYP-treated H9-GCaMP-CMs exhibited lower Ca^2+^ release amplitude (Figure 5B) and longer transient durations (Figure 5D). Interestingly, in addition to lower Ca^2+^ release amplitude, slower Ca^2+^ transient durations were observed to occur in H9-GCaMP-CMs treated with CYP at dose of 500 or 750 μmoL/L(Figures 5A,D). On day 8, compared with CON, H9-GCaMP-CMs treated with low, medium and high concentrations of CYP exhibited lower Ca^2+^ release amplitude, and longer time to peak and transient durations (Figures 5A–D). These observations indicated that CYP could induce abnormal electrophysiological and contractile alterations in cardiomyocytes, consistent with the findings of the RNA sequencing and clinical data.

### CYP Inhibited JPH2 Expression by Modulating the m6A Writer METTL3

**Figure 6:**
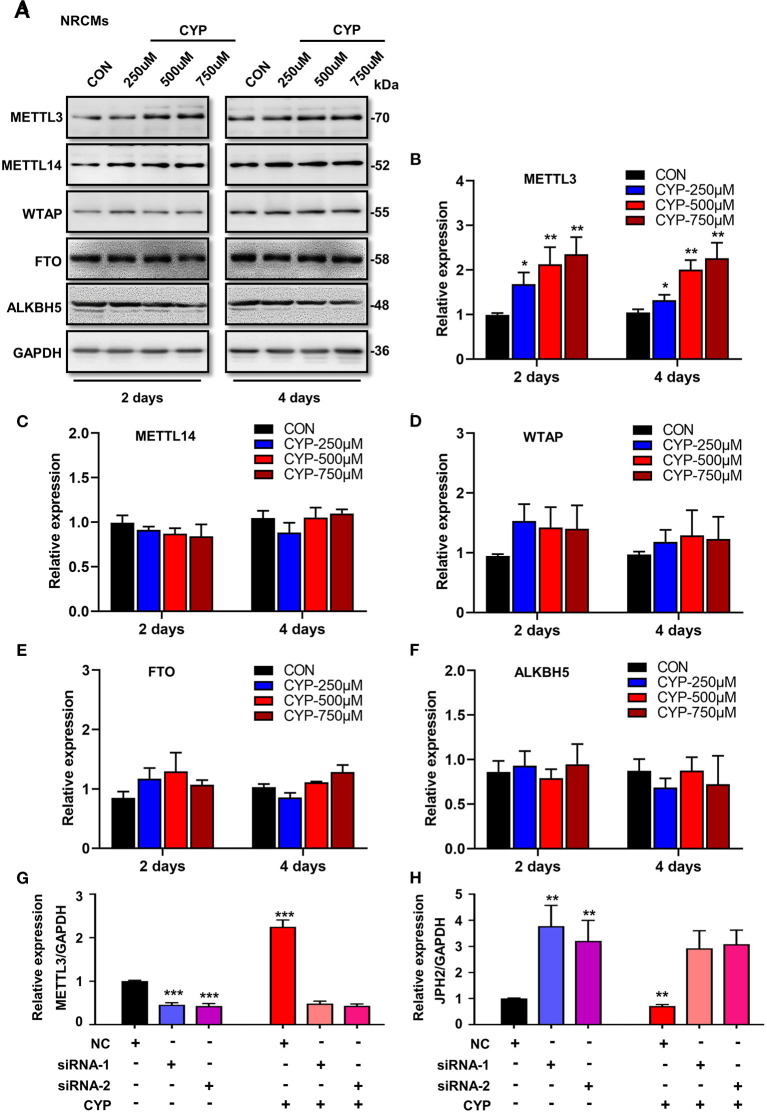
CYP induced the downregulation of JPH2 expression through upregulating METTL3 expression. **(A)** Western blot analyses of METTL3, METTL14, WTAP, FTO and ALKBH5 expression in NRCMs with or without CYP for 2 or 4 days. Quantitative analysis of protein levels of METTL3 **(B)**, METTL14 **(C)**, WTAP **(D)**, FTO **(E)**, and ALKBH5 **(F)** in NRCMs treated with CYP for 2 or 4 days. The data are shown as the mean ± SE of three experiments. *P < 0.05, **P < 0.01 vs. CON. Real-time PCR analysis of METTL3 **(G)** and JPH2 **(H)** expression in NRCMs transfected with si-METTL3, or NC sequences with/without CYP. CYP did not decrease the expression of JPH2 in METTL3-deficient NRCMs. The data are shown as the mean ± SE of three experiments. ** *P* < 0.01; *** *P* < 0.001 vs. negative control (NC).

To understand whether m6A RNA methylation plays an important role in the suppression effects of CYP on JPH2 expression in NRCMs, we further investigated the effect of CYP on m6A writers (METTL3, METTL14, and WTAP) and erasers (FTO, and ALKBH5) in CYP-treated cardiomyocytes. Intriguingly, exposing NRCMs to different doses of CYP (250, 500, and 750 umoL/L) for 2 or 4 days resulted in a significant increase in the expression of METTL3 (Figures 6A,B), whereas the expression of METTL14, WTAP, FTO, and ALKBH5 was not significantly altered (Figures 6A,C–F). Similar results were observed in rats treated with CYP. The RNA level of METTL3 increased in hearts (Supplementary Figure 6). These results suggested that CYP induces the m6A methylation of JPH2 mRNA through increasing METTL3 expression, leading to downregulation of JPH2 expression.

To investigate the biological effect of METTL3 on the reduction of JPH2 expression, we designed small interfering RNAs to silence METTL3 in NRCMs. Intriguingly, silencing METTL3 (Figure 6G) resulted in an increase in JPH2 expression (Figure 6H). Additionally, CYP induced JPH2 downregulation in NRCMs transfected with NC sequences. However, there was no reduction effect of CYP on JPH2 expression in si-METTL3 cardiomyocytes (Figure 6H). These data indicated that CYP decreased JPH2 expression by upregulating METTL3.

### Disruption of METTL3 Eliminated CYP-Induced Electrical Alterations of Cardiomyocytes

**Figure 7:**
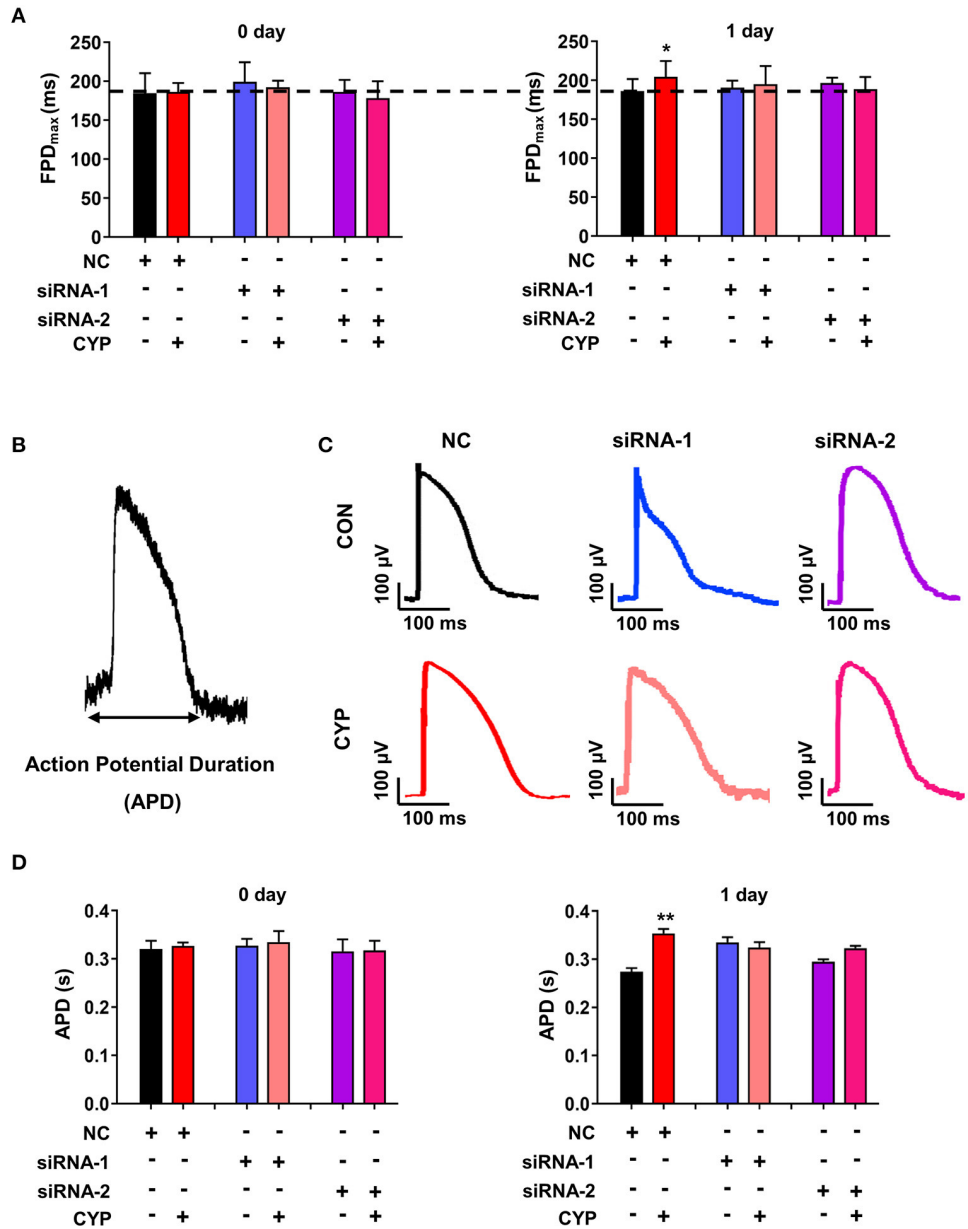
Disruption of METTL3 expression eliminated the increased field potential duration (FPD) and action potential duration (APD) induced by CYP in cardiomyocytes. **(A)** Silencing METTL3 expression eliminated the increase in FPD induced by CYP. **(B)** Schematic of APD by the MEA processing of cardiomyocytes. **(C)** Representative images of APD in NRCMs transfected with si-METTL3, or negative control (NC) sequences with/without CYP. **(D)** Quantification of APD in NRCMs transfected with si-METTL3 or NC sequences with/without CYP. Silencing METTL3 expression eliminated the increased APD induced by CYP. The data are shown as the mean ± SE of three experiments. * *P* < 0.05; ** *P* < 0.05 vs. NC.

To determine whether the disruption of METTL3 affects cardiac electrical and mechanical alterations in cardiomyocytes, we performed MEA in si-METTL3 and NC cardiomyocytes treated with CYP. Compared with NRCMs treated with NC, the FPD increased in NRCMs treated with CYP for 1 day (Figure 7A), whereas knock out of METTL3 significantly eliminated the increased FPD induced by CYP (Figure 7A). Similarly, the MEA results showed that the action potential duration (APD) (Figure 7B) was prolonged in NRCMs treated with NC after CYP treatment for 1 day (Figures 7C,D). However, the prolonged APD did not occur in si-METTL3 cardiomyocytes treated with CYP compared with the NRCMs treated with solvent control (Figures 7C,D). The above results demonstrated that the disruption of METTL3 eliminated the electrical alterations of cardiomyocytes induced by CYP.

## Discussion

**Figure 8:**
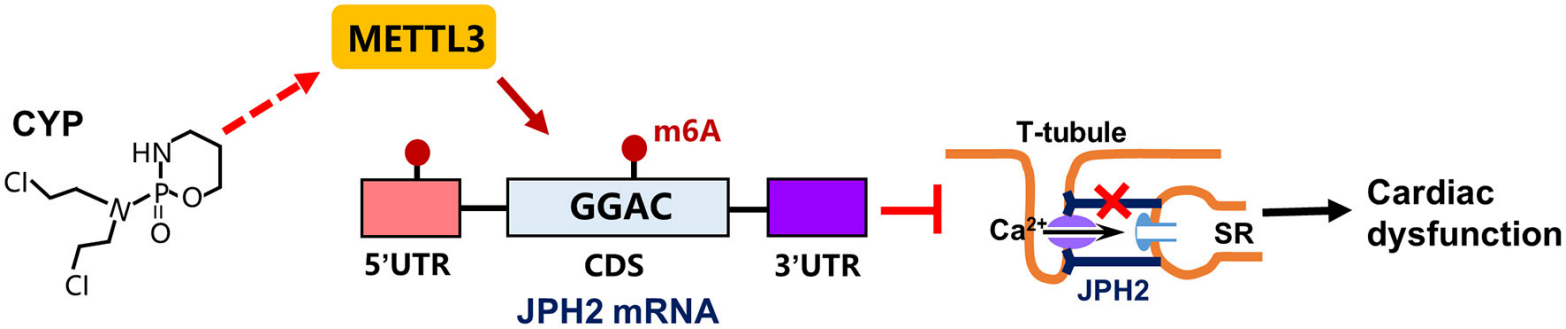
Schematic of CYP-induced cardiac electrical and mechanical alterations. CYP decreased JPH2 expression by upregulating METTL3 expression, leading to Ca^2+^ transient irregularities and cardiac dysfunction.

CYP is strongly correlated with cardiac electrical and contractile alterations (3, 40, 41). This study found that CYP was associated with QT prolongation, a decrease in E-C coupling efficiency, and cardiac contractile dysfunction. Specifically, our findings demonstrated that CYP induced RNA m6A modification by upregulating METTL3 expression and suppressing JPH2 expression (Figure 8). These results suggested novel therapeutic and preventive targets for CYP-induced cardiotoxicity.

CYP is widely used an antineoplastic and immunosuppressive agent. The cytotoxic effect of CYP is induced by its biologically active metabolites (4, 42). CYP decomposes into acrolein and phoramide mustard (43), which further produces an unstable cation that may attack guanine bases (4), resulting in methylated bases. These DNA methylations lead to mutations and pair mismatches linked with its therapeutic effects on tumor cells. In fact, alkylating agents cause various DNA alkylation lesions including base methylation (9), which also induce RNA methylation. In our study, total m6A levels significantly increased in NRCMs after CYP treatment. Our experimental results showed that the m6A writer METTL3 significantly increased in cardiomyocytes treated with CYP, leading to an increase in m6A methylation of JPH2 mRNA. Promotion of the upregulation of METTL3 expression by CYP needs further exploration, however, the results suggested that RNA methylation played an important role in CYP-induced cardiotoxicity.

Previous study highlighted that CYP induced cardiac apoptosis when administered at a high dose (44), because the metabolite of CYP acrolein could promote the formation of reactive oxygen species (ROS) (45, 46). Hence, some studies have aimed to inhibit reactive oxygen-generators and regenerate other antioxidants that could prevent or treat CYP-induced acute cardiotoxicity (47). In this study, no myocardial death occurred in rats after treatment with CYP. We also observed no significant effect on the viability of cardiomyocytes in NRCMs treated with CYP at high concentrations. However, ANP and BNP both increased in cardiomyocytes treated with CYP, consistent with the findings of a previous study that showed CYP could induce cardiac hypertrophy (44). In this study, there was no obvious ventricular wall thickening in the ultrasound results owing to the short duration of CYP treatment in rats and administration being performed only once. However, increased ANP and BNP levels suggest that the molecular pathological changes may precede structural changes and the prolonged CYP treatment is required for organic changes to occur. Meanwhile, we found cardiac electrical alterations and decreased E-C coupling efficiency in rats after CYP administration. Although FS and LVEF did not decrease to heart failure in rats treated with CYP, these results were a 1-time consequence of CYP treatment with normal doses. Although prolonged QT and QTc interval, as well as E-C coupling time courses would recover after 3 days of administration, our results have implications for some patients with potential risk of ECG abnormalities during therapy for cancer and immune diseases. Interestingly, our data showed that CYP induced cardiac prolonged QT intervals and electromechanical coupling time courses accompanied by the downregulation of JPH2 expression. Calpain hydrolyzes JPH2 at the protein level (48), but CYP-induced decrease in JPH2 expression initiated from the RNA level in this study. To verify whether CYP-induced downregulation of JPH2 expression is mediated by miR-24 (31) and miR-331 (35), we further explored the effect of CYP on the biogenesis of the two miRNAs. There were no increases in the effect of CYP on miR-24 and miR-331, suggesting other regulatory mechanisms for JPH2. Interestingly, our subsequent results showed that m6A RNA methylation was associated with decreased expression of JPH2. These results suggested that the increase in m6A of JPH2 mRNA is a novel mechanism in CYP-induced cardiotoxicity.

To investigate the mechanisms underlying of CYP-induced cell toxicity, we performed RNA sequencing to explore the potential targets and pathways. Our results showed that these DEGs were enriched in the biological process categories of leukocyte, lymphocyte and T cell-mediated immunity, which corresponded to a recent study that CYP actively recruited macrophages into the bone marrow and eliminated drug-resistant malignant tumor cells (49). However, whether the positive regulation of immunity induces cardiac injury requires further study. These DEGs enriched in molecular function categories of phosphatidylinositol bisphosphate, phosphatidylinositol-4,5-bisphophate binding, ATPase activity, and metal ion transmembrane transporter activity were associated with reduced ATP production and failure of Ca^2+^ transient in cardiomyocytes. According to this finding, the KEGG analysis showed that these DEGs were involved in the inflammation and calcium signaling pathways. Interestingly, we observed that cAMP signaling and the GnRH pathway were closely associated with the calcium signal and cardiac contraction (50, 51). The calcium signaling pathway was enriched in upregulated DEGs from MeRIP sequencing. In subsequent exploration of the effect of CYP on calcium signal, we found that CYP induces lower calcium release amplitude, and longer time to peak and transient durations. CYP-treated H9-GCaMP-CMs even exhibited lower calcium transient durations. These results are consistent with the adverse cardiac phenotype caused by CYP, suggesting that the calcium signaling pathway plays an important role in CYP-induced cardiotoxicity. Notably, the expression of JPH2, a key regulator for the Ca^2+^ influx and E-C coupling in cardiomyocytes (31, 52), significantly reduced after CYP treatment. Because decreased JPH2 is reportedly associated with atrial fibrillation (33) and arrhythmias (34), consistent with CYP-induced cardiotoxicity events, CYP-induced cardiac electrical and mechanical alterations may be closely related to the downregulation of JPH2 in this study. However, we cannot exclude other potential genes that play roles in regulating the process, such as paralemmin 2 (Palm-2), which upregulated m6A peaks and downregulated gene expression, was associated with cAMP-PKA signaling pathway, which has a strong influence on intracellular cation concentrations in the heart tissue or cardiomyocytes (53).

Previous epidemiological studies have suggested that prolonged QT intervals are closely associated with abnormal sodium, and potassium channels (54). However, the relationships between calcium ion binding protein imbalance and the pathological mechanism of QT prolongation are unknown. Recent studies have shown that Ca^2+^ binding proteins such as calmodulin (55, 56), and triadin (57), are associated with the long QT syndrome. These studies suggested that calcium plays an important role in the pathogenesis of cardiomyocyte repolarization and QT interval prolongation (58). JPH2 is the key regulatory protein that maintains a normal distance between LCCs and RyRs, which are important structures for Ca^2+^ release and recovery in cardiomyocytes. Moreover, a recent study demonstrated that the N-terminal part of JPH2 could bind and interact with caveolin-3 (59), which is a critical mediator for fixing LCCs on caveolar membrane in the plasma membrane and associated with long QT syndrome (60). Caveolin-3 is an important member of muscle-specific structural proteins of caveolae, which are also localized in T-tubules (61). These studies suggested that JPH2 interacts with caveolin-3 to mediate the junctional membrane complexes and Ca^2+^-induced Ca^2+^ release in the cardiomyocytes (59). Although abnormal JPH2 expression decreases the fixation with caveolin-3, leading to disruption of the normal junctional membrane complexes and efficient Ca^2+^ transient, it may positively affect the QT interval. In this study, CYP induced the downregulation of JPH2 expression, resulting in increased FPD and APD in cardiomyocytes, which would be eliminated by silencing METTL3. Our results suggested that JPH2 aberration is closely related to the long QT syndrome. However, clinical data is warranted to determine whether the absence of JPH2 leads to the prolonged QT interval in future studies.

Despite these encouraging results, it is necessary to point out the limitations of this study. Silencing METTL3 increases the JPH2 expression, and JPH2 is not further downregulated in si-METTL3 NRCMs after CYP treatment. It is significant to use METTL3 knockout transgenic mice to verify whether CYP induced cardiac electrical and mechanical alterations by increasing m6A levels. Additionally, there are m6A methylation sites in both the 5'UTR and CDS of JPH2 mRNA, and the m6A methylation modification sites that regulate the expression of JPH2 need to be further clarified. Furthermore, the m6A levels and JPH2 expression abnormalities in CYP-induced cardiotoxicity should be confirmed in the clinic in future studies.

In summary, our results indicated that CYP-induced cardiac electrical and mechanical alterations and Ca^2+^ dyshomeostasis are associated with m6A methylation modifications and decreased JPH2. Our study found that CYP increased RNA m6A levels by altering METTL3 expression. Furthermore, decreased JPH2 expression plays an important role in CYP-induced cardiac electrical and mechanical alterations by blocking Ca^2+^ influx between transverse tubules and sarcoplasmic reticulum. Our findings demonstrated that RNA m6A methylation is a potential therapeutic intervention for CYP-induced cardiotoxicity.

## Data Availability Statement

The datasets presented in this study can be found in online repositories. The names of the repository/repositories and accession number(s) can be found below: https://www.ncbi.nlm.nih.gov/geo/query/acc.cgi?acc=GSE184294.

## Ethics Statement

The animal study was reviewed and approved by the Ethics Committee of Peking University Health Science Centre (LA2021004).

## Author Contributions

MX and MZ conceived and designed the experiments. MZ and YL performed experiments and acquired data. YS, SZ, CH, XL, and FL provided materials. MZ, YL, and ZH performed data analysis. MZ, YL, and MX wrote the manuscript. All authors contributed to the article and approved the submitted version.

## Funding

This study was supported by the grants from National Natural Science Foundation of China (81625001 and 81900315) and the National Key Research & Development Program of China (2018YFC1312700 and 2018YFC1312701).

## Conflict of Interest

The authors declare that the research was conducted in the absence of any commercial or financial relationships that could be construed as a potential conflict of interest.

## Publisher's Note

All claims expressed in this article are solely those of the authors and do not necessarily represent those of their affiliated organizations, or those of the publisher, the editors and the reviewers. Any product that may be evaluated in this article, or claim that may be made by its manufacturer, is not guaranteed or endorsed by the publisher.
