# Supplementary material for: The Role of METTL3-Mediated N6-Methyladenosine (m6A) of JPH2 mRNA in Cyclophosphamide-Induced Cardiotoxicity
Source: Front Cardiovasc Med. 2021 Nov 8;8:763469. doi: 10.3389/fcvm.2021.763469 (PMC8606687; doi:10.3389/fcvm.2021.763469)
Supplement: Supplementary file 1 [file Data_Sheet_1.DOCX]

**SUPPLEMENTARY METHODS.**

**Cell viability assays**

﻿Cell viability was examined by a Cell counting kit-8 (CCK8) assay (Dojindo, Japan) CCK8 assay according to the protocol of the manufacturer. Cells were seeded in quintuplicate in each well of 96-well culture plates and treated with CYP (S2057) (Selleckchem, USA) at different concentration (0 umoL/L, 62.5 umoL/L, 125 umoL/L, 250umoL/L, 500 umoL/L, and 1000 umoL/L) and 10 ml of CCK8 solution were added into each well at 48 hours. The absorbancy A450 was measured to evaluate cell viability.

**RNA** **sequencing and bioinformatics analysis**

Total RNA was obtained from NRCMs using TRIzol reagent (Invitrogen). RNA library construction and sequencing was performed on the BGISEQ-500 platform at Beijing Genomics Institute (BGI, Beijing, China) as described previously (1). The gene expression levels were measured using the FPKM method. Differentially expressed genes (DEGs) were identified under a threshold of fold change ≥1.5 and FDR≤0.01, and then were used for gene ontology (GO) enrichment and Kyoto Encyclopedia of Genes and Genomes (KEGG) analysis.

**REFERENCES**

1. Wang Z, Li G, Wu Q, Liu C, Shen J, and Yan W. Microcystin-LR exposure induced nephrotoxicity by triggering apoptosis in female zebrafish. *Chemosphere*. (2019) 214:598-605. doi: 10.1016/j.chemosphere.2018.09.103.

**SUPPLEMENTARY FIGURE.**

**
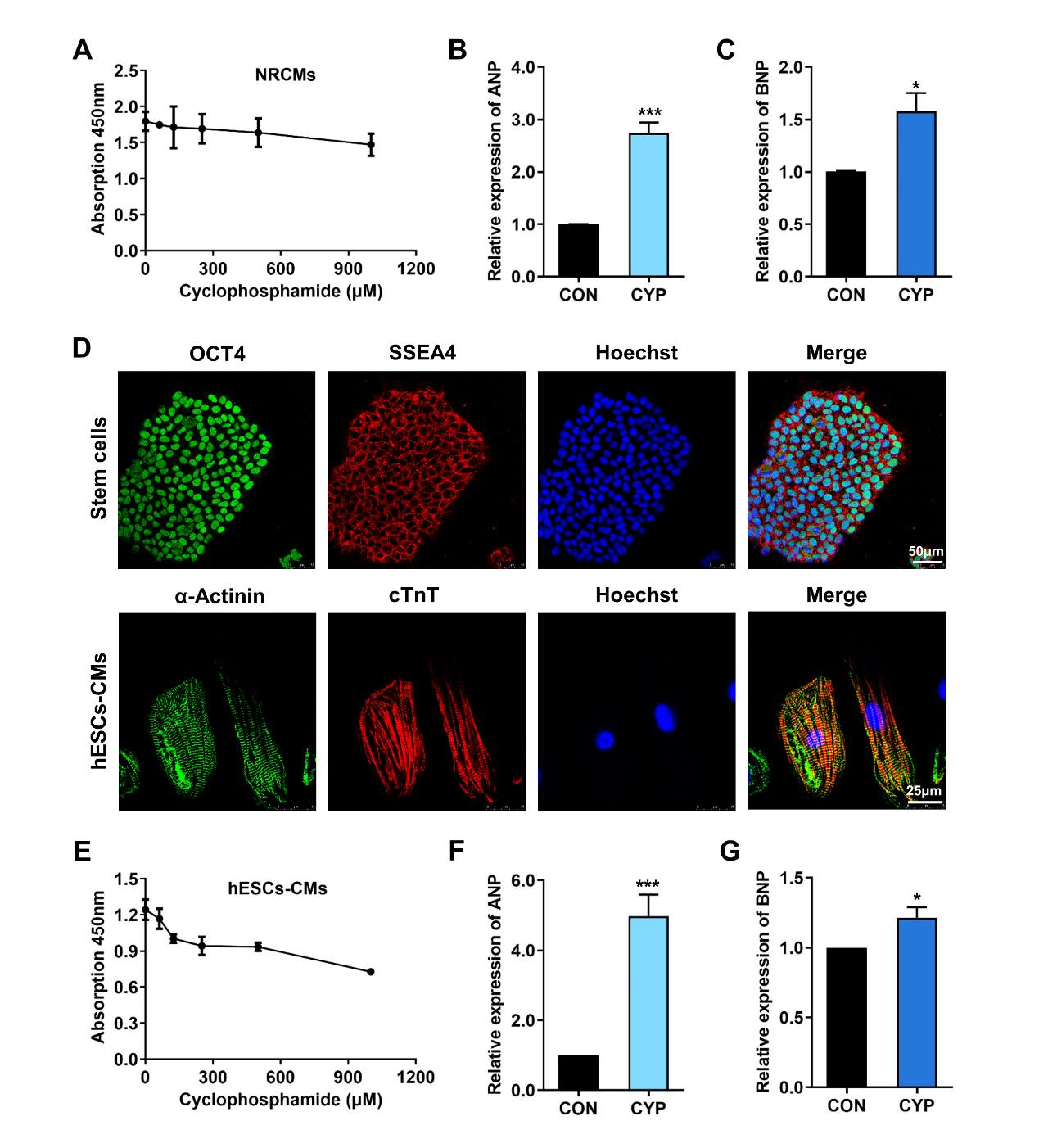
**

**Supplementary Figure 1|** The effect of CYP treatment on cardiomyocytes viability. **(A)** CCK-8 assay for NRCMs with different concentration of CYP. Real-time PCR analysis of ANP **(B)** and BNP **(C)** expression in NRCMs treated with CYP or solvent control (CON). **(D)** Cardiomyocytes were differentiated from human embryonic stem cells (hESCs). Representative immunofluorescence staining of hESCs for markers of pluripotent stem cells, including OCT4 (green) and SSEA4 (red); scale bar=50 μm. Immunofluorescent staining for α-actinin (green) and TNNT2 (red) to demonstrate hESCs-CMs; scale bar=25 μm. **(E)** CCK-8 assay for hESCs-CMs with different concentration of CYP. Real-time PCR analysis of ANP **(F)** and BNP **(G)** expression in hESCs-CMs treated with CYP or solvent control. The data are shown as the mean ± SE from three separate experiments. **P* ˂ 0.05, ****P* ˂ 0.001 vs. CON.


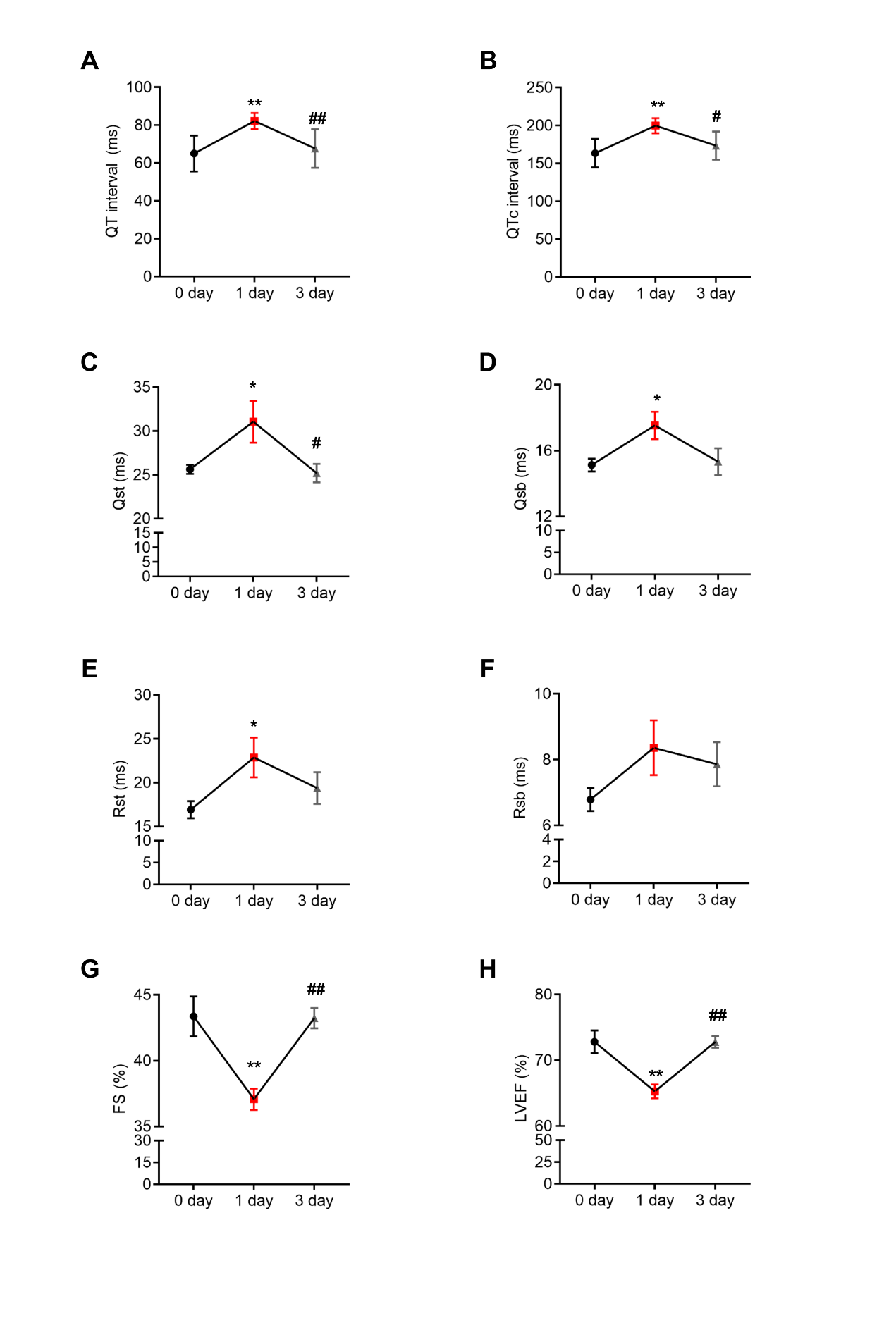


**Supplementary Figure 2|** Changes of QT intervals, cardiac electromechanical coupling and cardiac function in rats with CYP treatment. Electrocardiogram recording showing the changes of QT intervals **(A)** and QTc **(B)** in rats treated with CYP at days 1 and 3. The changes of Qsb **(C)**, Qst **(D)**, Rsb **(E)**, and Rst **(F)** in CYP-treated rats for 1 or 3 days compared to 0 day. The changes of FS **(G)** and LVEF **(H)** in rats subjected to CYP at days 0–3, The data are represented as mean ± SE, n = 6. **P* < 0.05, ***P* < 0.01 vs. 0 day; ^#^*P* < 0.05, ^##^*P* < 0.01 vs. 1 day.


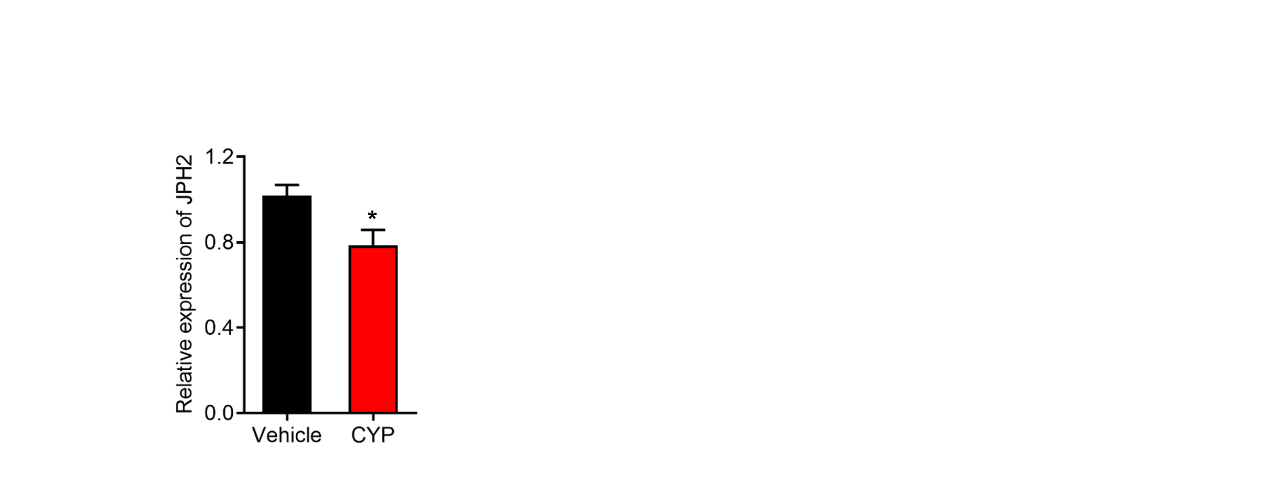


**Supplementary Figure 3|** Quantitative real-time PCR analysis of RNA level of JPH2 in the heart tissues of rats with vehicle or CYP treatment. The data are represented as mean ± SE, n = 6. **P* < 0.05 vs. CON.


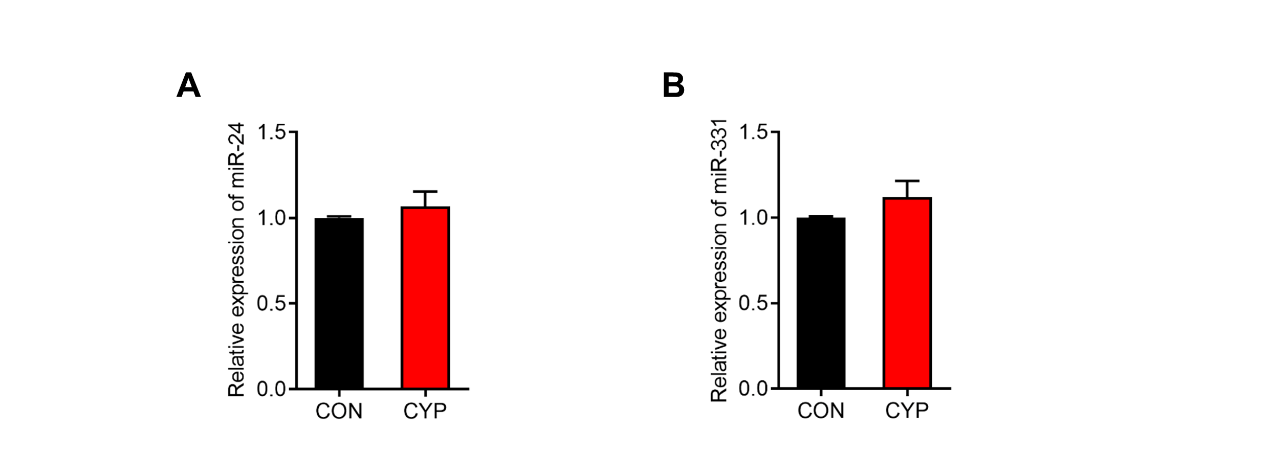


**Supplementary Figure 4|** The effects of CYP on the miR-24 and miR-331 expression in cardiomyocytes. Real-time PCR analysis of miR-24 **(A)** and miR-331 **(B)** expression in NRCMs treated with CYP or solvent control (CON). The data are shown as the mean ± SE from three separate experiments.

**
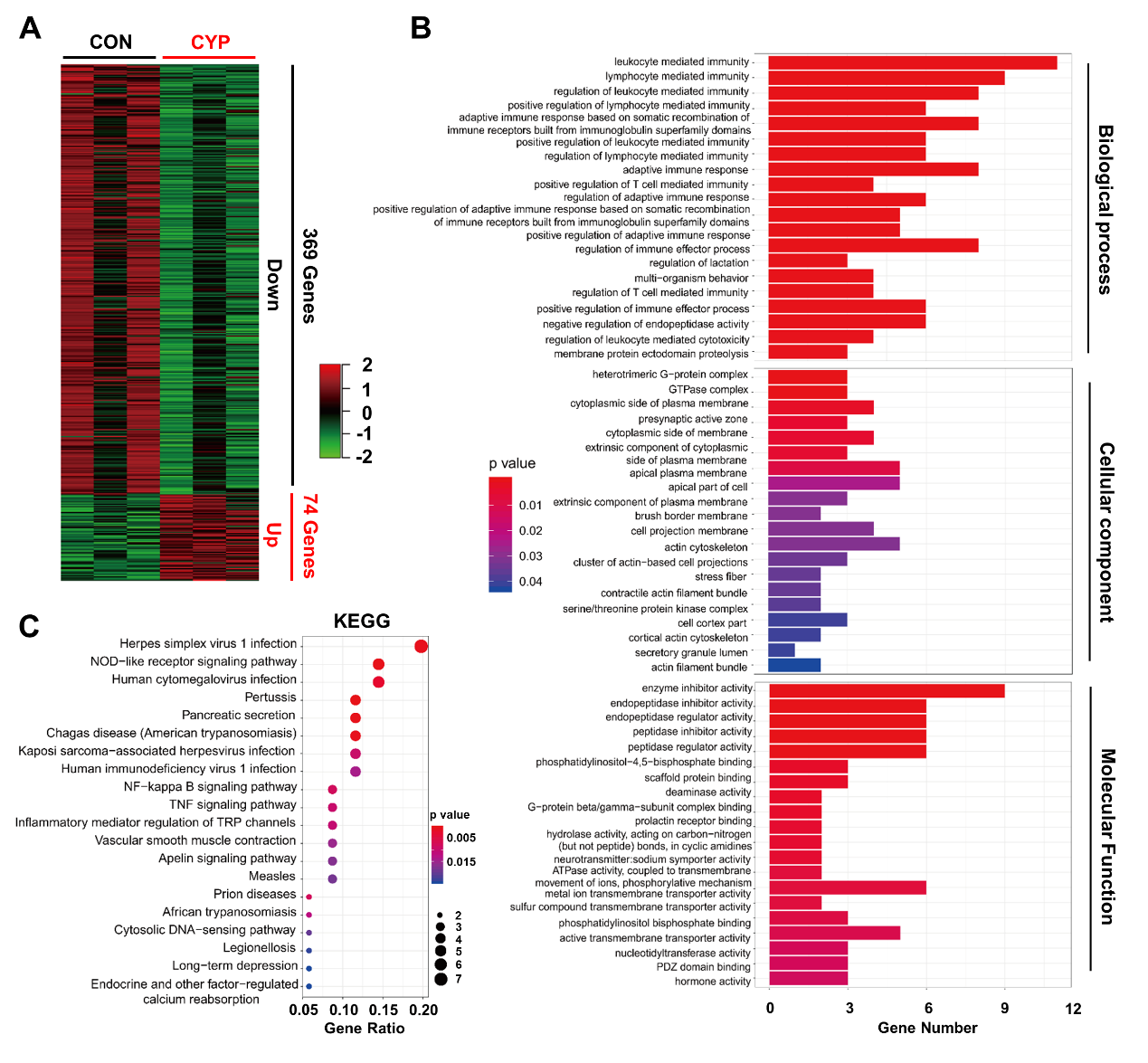
**

**Supplementary Figure 5|** Analysis of transcriptional characteristics of NRCMs with or without CYP. **(A)** Heatmap shows hierarchical clustering of DEGs with or without CYP treatment. Values are row-scaled to show relative expression. Representative up-regulated (red) and down-regulated (green) genes are presented. **(B)** Gene ontology (GO) term categorization and distribution of DEGs. GO terms were processed and categorized under three categories (cellular component, molecular function, and biological process). **(C)** These DEGs are enriched in detail KEGG pathway figure.

**
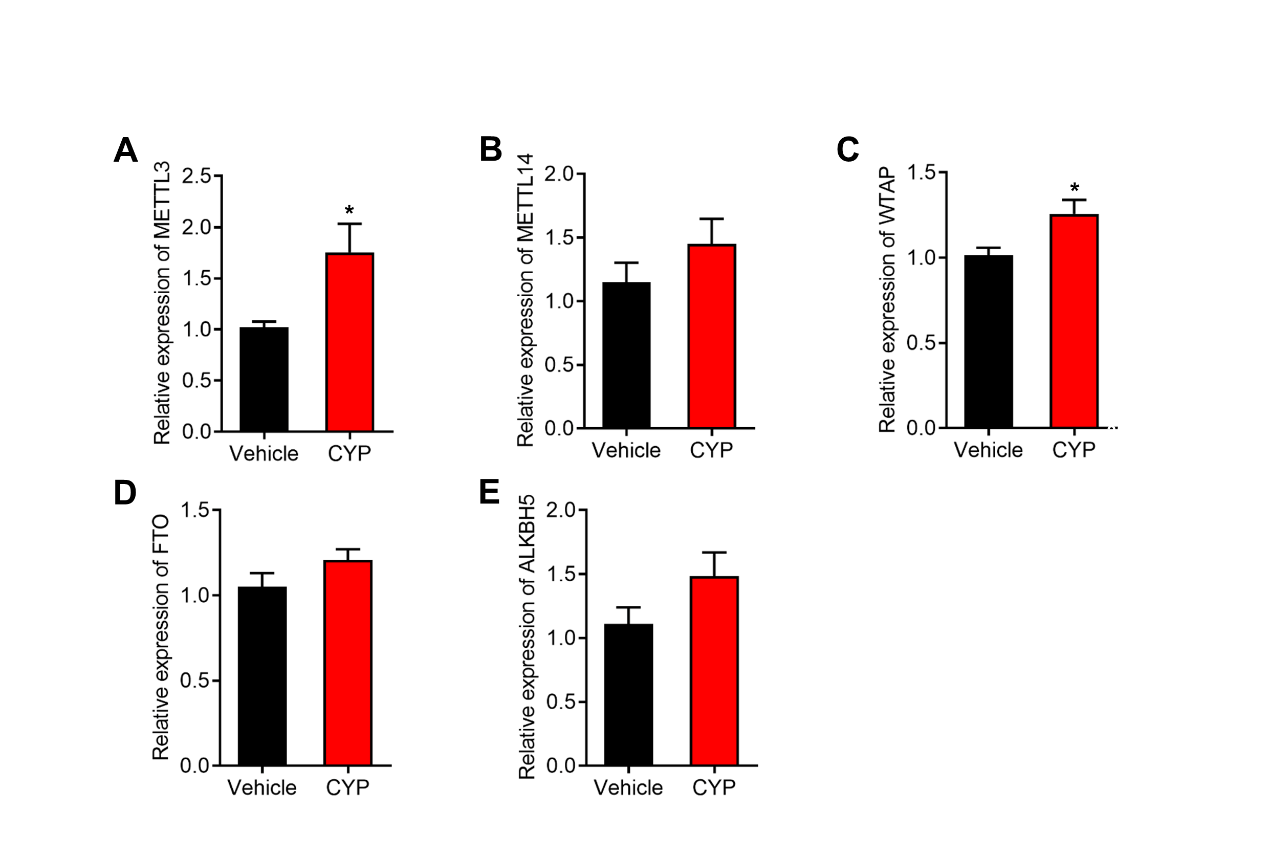
**

**Supplementary Figure 6|** Quantitative real-time PCR analysis of RNA level of methyltransferases in the heart tissues of rats with vehicle or CYP treatment. Quantitative real-time PCR analysis of METTL3 **(A)**, METTL14 **(B)**, WTAP **(C)**, FTO **(D)** and ALKBH5 **(E)** in the heart tissues of rats treated with vehicle or CYP for 1 day. The data are represented as mean ± SE, n = 6. **P* < 0.05 vs. vehicle.

| **SUPPLEMENTARY TABLE.**  **Supplementary Table 1\|** Antibodies for immunofluorescence and western blot in this study | | |
| --- | --- | --- |
| primary antibodies | Dilution | Company/Catalog |
| OCT4 | 1:100 | Santa Cruz/Sc-5279 |
| SSEA4 | 1:100 | Santa Cruz/Sc-21704 |
| α-Actinin | 1:100 | abcam/ab137346 |
| cTnT | 1:100 | abcam/ab45932 |
| METTL3 | 1:2000 | Proteintech/15073-1-AP |
| ALKBH5 | 1:1000 | Proteintech/16837-1-AP |
| METTL14 | 1:500 | abcam/ab98166 |
| FTO | 1:1000 | abcam/ab92821 |
| GAPDH | 1:10000 | Proteintech/10494-1-AP |

| **Supplementary Table 2\|** Primers and siRNAs used in this study | |
| --- | --- |
| Name | Sequence (5’-3’) |
| Rno-ANP-Forward | CTCCGATAGATCTGCCCTCTTGAA |
| Rno-ANP-Reverse | GGTACCGGAAGCTGTTGCAGCCTA |
| Rno-BNP-Forward | TTTCCTTAATCTGTCGCCGCT |
| Rno-BNP-Reverse | TGCATCGTGGATTGTTCTGGA |
| Rno-Kif21b-Forward | CGACGAGTCCTGGCCATAAA |
| Rno-Kif21b-Reverse | CTAACTTCAGGGGTGGGCTG |
| Rno-Myo9a-Forward | CTCCCGGACTCACCTCAGTA |
| Rno-Myo9a-Reverse | TGCAAGGTGCAAACACGATG |
| Rno-Kif1b-Forward | AGTGGAGGTCACCGAGTCAT |
| Rno-Kif1b-Reverse | TAGTGGCTGGAACTGGTCTG |
| Rno-Mid1-Forward | ACCTGTTAGGCTGACCTGAG |
| Rno-Mid1-Reverse | GGGAGCTGGAAAAGGCAAAC |
| Rno-Trps1-Forward | GCACCGGCATGCAGAGTAAAG |
| Rno-Trps1-Reverse | TGTGTTCCTCCTTCGTGTTCA |
| Rno-Xiap-Forward | CTAATCGAGGGCTGCTCGG |
| Rno-Xiap-Reverse | CCAGTCCTAACTAACCGCCG |
| Rno-Inhba-Forward | ATCCGTCTGTTTCAGCAGCA |
| Rno-Inhba-Reverse | CGCACATCCAGGGAACTCTT |
| Rno-Ddx6-Forward | CCTGGGTGGTAGGACTCGG |
| Rno-Ddx6-Reverse | TCAGGCAAGCACCTGTAAGT |
| Rno-Palm2-Forward | TGGCTTACGCAATGAACCCT |
| Rno-Palm2-Reverse | TTTGGGGGAGCATGGAAACA |
| Rno-JPH2-Forward | AGGCGGGTGCCAAGAAGAAG |
| Rno-JPH2-Reverse | CGATGTTCAGCAGGATCACCA |
| Rno-METTL3-Forward | CTTTAGCATCTGGTCTGGGCT |
| Rno-METTL3-Reverse | CCTTCTTGCTCTGCTGTTCCT |
| Rno-METTL14-Forward | GCAGAAACCTACGCGTCCTA |
| Rno-METTL14-Reverse | CACCACGGTCAGACTTGGAT |
| Rno-WTAP-Forward | CTCGCCTCGTCTCTTCTGG |
| Rno-WTAP-Reverse | CATCTTGTACCCCGAGACGC |
| Rno-FTO-Forward | ACTGGTTTTCCGAGAGGCTG |
| Rno-FTO-Reverse | GTGAGCACGTCTTTGCCTTG |
| Rno-ALKBH5-Forward | ACCACCAAACGGAAGTACCAG |
| Rno-ALKBH5- Reverse | TCATCCTGGCTGAAGAGACG |
| Rno-GAPDH-Forward | TCCCTCAAGATTGTCAGCAA |
| Rno-GAPDH-Reverse | AGATCCACAACGGATACATT |
| Hsa-ANP-Forward | ACAATGCCGTGTCCAACGCAGA |
| Hsa-ANP-Reverse | CTTCATTCGGCTCACTGAGCAC |
| Hsa-BNP-Forward | TCTGGCTGCTTTGGGAGGAAGA |
| Hsa-BNP-Reverse | CCTTGTGGAATCAGAAGCAGGTG |
| Hsa-GAPDH-Forward | CGACCACTTTGTCAAGCTCA |
| Hsa-GAPDH-Reverse | AGGGGTCTACATGGCAACTG |
| METTL3-siRNA-1 | UCACAAACCAGAUGAAAUATT |
|  | UAUUUCAUCUGGUUUGUGATT |
| METTL3-siRNA-2 | CUAAGGAACAGCAGAGCAATT |
|  | UUGCUCUGCUGUUCCUUAGTT |
| Negative Control | UUCUCCGAACGUGUCACGUTT |
|  | ACGUGACACGUUCGGAGAATT |
